# O6-Methylguanine-DNA Methyltransferase (MGMT) Promoter Methylation-Guided Irinotecan-Based Therapy in Metastatic Colorectal Cancer: A Case Report

**DOI:** 10.7759/cureus.113070

**Published:** 2026-07-21

**Authors:** Pramod K Julka, Deepika Arya, Afnan Bhasir, Sanjay Gupta

**Affiliations:** 1 Max Institute of Cancer Care, Max Smart Super Speciality Hospital, Saket, New Delhi, IND; 2 Clinical Research, Catalyst Clinical Services Pvt. Ltd., New Delhi, IND

**Keywords:** circulating tumor dna (ctdna), guardant360® liquid, metastatic colorectal cancer, mgmt promoter methylation, precision oncology

## Abstract

The management of metastatic colorectal cancer (mCRC) has evolved toward precision oncology, integrating molecular biomarkers to guide therapeutic selection. While RAS status remains a key determinant of response to anti-epidermal growth factor receptor (anti-EGFR) therapy, emerging alterations in DNA repair pathways, including O^6^-methylguanine-DNA methyltransferase (MGMT) promoter methylation, may reveal additional, biologically relevant therapeutic vulnerabilities. We describe a 45-year-old female patient with a prior history of breast carcinoma who presented with metastatic high-grade colorectal adenocarcinoma involving the sigmoid colon and liver. Immunohistochemistry (IHC) confirmed colorectal origin. Comprehensive molecular profiling demonstrated KRAS wild-type status, microsatellite stability, low tumor mutational burden (TMB), and canonical alterations in adenomatous polyposis coli (APC) and tumor protein p53 (TP53). First-line capecitabine and oxaliplatin (CAPOX) resulted in disease progression following initial disease stabilization. Subsequent circulating tumor DNA (ctDNA) analysis identified MGMT promoter methylation, suggesting impaired DNA repair capacity. In the absence of actionable genomic targets, second-line therapy with capecitabine and irinotecan (CAPIRI) and cetuximab was initiated. This approach yielded a durable partial response, with significant reduction in hepatic metastatic burden and the preservation of functional status. While established treatment sequencing remains the cornerstone of mCRC management, ctDNA-detected MGMT promoter methylation offers an additional biologically informed stratification marker, suggesting underlying DNA repair deficiency and potential sensitivity to irinotecan-based therapy.

## Introduction

Colorectal cancer (CRC) remains a major global health challenge and is one of the leading causes of cancer-related morbidity and mortality worldwide [1]. Approximately half of all patients develop metastatic colorectal cancer (mCRC), a stage associated with poor long-term survival that necessitates systemic therapy [2]. Combination chemotherapy regimens incorporating fluoropyrimidines with oxaliplatin or irinotecan, including folinic acid, fluorouracil, and oxaliplatin (FOLFOX), folinic acid, fluorouracil, and irinotecan (FOLFIRI), and capecitabine and oxaliplatin (CAPOX), constitute the cornerstone of first-line treatment. Among these, CAPOX has gained widespread acceptance owing to its efficacy comparable to infusional fluoropyrimidine-based regimens while offering the convenience of oral capecitabine administration.

The therapeutic landscape of mCRC has evolved substantially with the advent of precision oncology. Molecular biomarkers, including RAS mutation and microsatellite instability (MSI) status, have become integral to therapeutic decision-making by identifying patients most likely to benefit from targeted therapies and immune checkpoint inhibitors, thereby facilitating increasingly individualized treatment strategies [3-8]. Complementing this paradigm, circulating tumor DNA (ctDNA)-based liquid biopsy has emerged as a powerful, minimally invasive approach for comprehensive genomic and epigenomic profiling. Platforms such as Guardant360® Liquid (Guardant Health, Palo Alto, CA, USA), a ctDNA-based next-generation sequencing assay, enable real-time molecular characterization from peripheral blood and are particularly valuable in metastatic disease when tumor tissue is unavailable or insufficient. In addition to informing treatment selection, ctDNA analysis supports longitudinal response assessment and the detection of minimal residual disease (MRD), further enhancing precision-guided therapeutic decision-making [9].

Among emerging molecular biomarkers, O^6^-methylguanine-DNA methyltransferase (MGMT) has attracted considerable attention because of its critical role in DNA repair and its potential therapeutic implications. Epigenetic silencing through promoter methylation results in loss of MGMT expression and is observed in approximately 30-40% of mCRC cases [10,11]. Although its prognostic significance remains uncertain, accumulating evidence suggests that MGMT promoter methylation may serve as a predictive biomarker of enhanced sensitivity to irinotecan and other DNA-damaging agents, offering a promising avenue for treatment personalization [12,13]. Herein, we present the case of a patient with metastatic high-grade adenocarcinoma of lower gastrointestinal origin and a prior history of breast cancer, in whom comprehensive ctDNA-based molecular profiling identified MGMT promoter methylation. This case highlights the diagnostic complexities of advanced disease and illustrates the potential clinical value of comprehensive molecular profiling in informing individualized therapeutic strategies and optimizing patient outcomes.

## Case presentation

**Figure 1:**
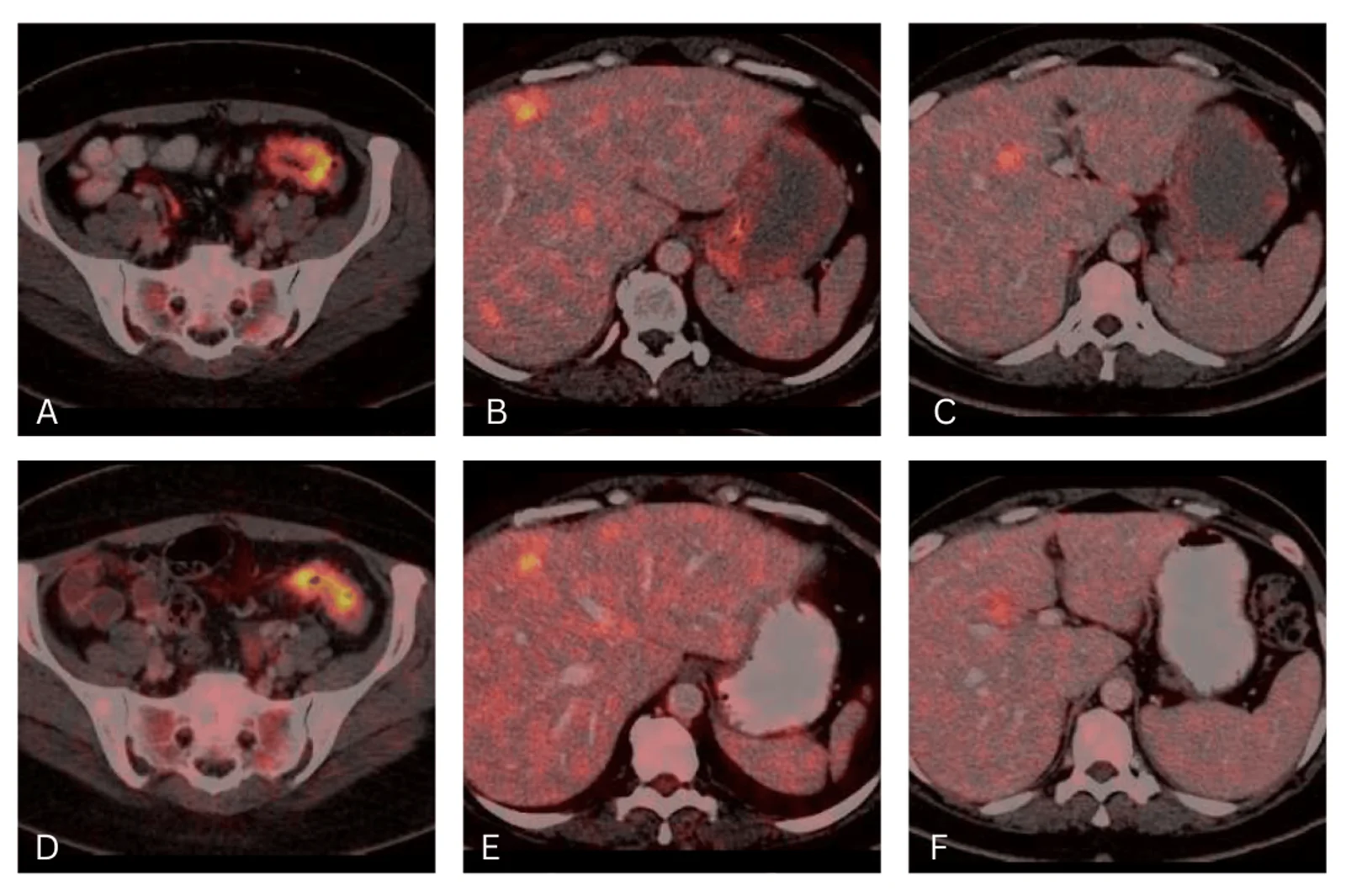
Baseline imaging and response to first-line therapy (CAPOX) (A) Circumferential thickening of the sigmoid colon, suggestive of a primary malignancy (December 2024). (B-C) Multiple hepatic metastases (December 2024). (D-F) Response after four cycles of first-line CAPOX chemotherapy, showing stable disease (April 2025). CAPOX: capecitabine and oxaliplatin

A 45-year-old female patient with a prior history of right breast carcinoma, treated with modified radical mastectomy followed by adjuvant radiotherapy and trastuzumab in 2012, presented in December 2024 with complaints of left-sided pelvic pain. On initial clinical evaluation, the patient had an Eastern Cooperative Oncology Group (ECOG) performance status of 1. Radiological assessment, including contrast-enhanced computed tomography (CECT) and positron emission tomography-computed tomography (PET-CT), revealed circumferential thickening of the sigmoid colon, suggestive of a primary malignancy (Figure 1A).

Additional findings included multiple hepatic metastases (Figure 1B-1C), pericolonic lymphadenopathy, perilesional fat stranding, and possible involvement of adjacent structures, including the urinary bladder. Subsequent sigmoidoscopy demonstrated a large, irregular annular lesion in the sigmoid colon. Histopathological evaluation of a liver biopsy confirmed metastatic high-grade adenocarcinoma, while concurrent breast biopsy findings were consistent with a benign fibroadenoma. Immunohistochemistry (IHC) demonstrated tumor cells positive for cytokeratin 20 (CK20), caudal type homeobox 2 (CDX2), and special AT-rich sequence-binding protein 2 (SATB2) and negative for cytokeratin 7 (CK7), GATA-binding protein 3 (GATA3), paired box gene 8 (PAX8), and Wilms tumor protein 1 (WT1), consistent with a colorectal primary.

**Figure 2:**
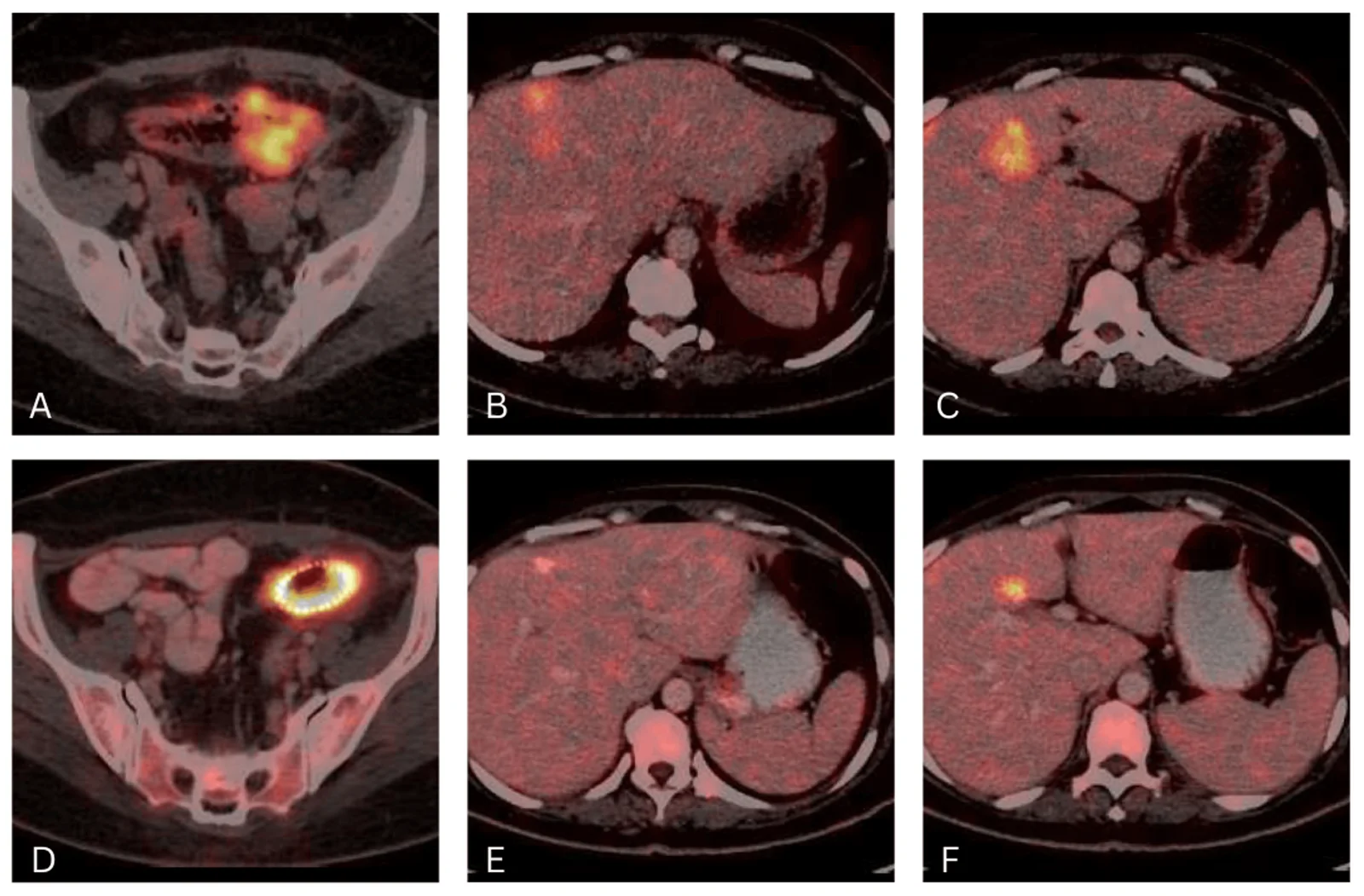
Disease progression on CAPOX and response to second-line therapy (CAPIRI + cetuximab) (A-C) Response after eight cycles of CAPOX demonstrating radiological disease progression (July 2025). (D-F) Response after six cycles of second-line CAPIRI plus cetuximab, demonstrating partial response with reduction in hepatic metastatic burden and no new lesions (January 2026). CAPIRI: capecitabine and irinotecan

Further molecular profiling using next-generation sequencing (FoundationOne® platform, Roche Diagnostics, Mannheim, Germany) revealed programmed death-ligand 1 (PD-L1) expression of approximately 1%, microsatellite stable (MSS) status, and homologous recombination deficiency (HRD)-negative status. The tumor was KRAS wild-type and harbored genomic alterations in adenomatous polyposis coli (APC), MYC proto-oncogene, bHLH transcription factor (MYC), ADP-ribosylation factor-related protein 1 (ARFRP1), Bcl-2-like protein 1 (BCL2L1), and tumor protein p53 (TP53). Based on the clinical, pathological, and molecular findings, a diagnosis of metastatic high-grade colorectal adenocarcinoma was established. The patient was initiated on first-line chemotherapy with the CAPOX regimen administered every three weeks. CAPOX regimen was selected following shared decision-making with the patient because of the convenience of oral capecitabine administration and the patient's preference to avoid continuous infusional fluoropyrimidine-based therapy due to concerns regarding potential treatment-related toxicity. Although the tumor was KRAS wild-type, anti-epidermal growth factor receptor (anti-EGFR) therapy was deferred in the first-line setting based on patient preference, with the intention of reserving this biomarker-directed treatment option for a subsequent line of therapy if clinically indicated. Interim response assessment with PET-CT after four cycles demonstrated stable disease (Figure 1D-1F), and treatment was continued for an additional four cycles. However, follow-up imaging after the completion of eight cycles revealed radiological disease progression (Figure 2A-2C).

In view of disease progression, ctDNA analysis was performed using the Guardant360® Liquid platform to further characterize the evolving molecular profile. The analysis identified APC frameshift mutations and a TP53 R248W mutation, along with MGMT promoter methylation. Variant allele fractions ranged from 2.3% to 5.3%, with an estimated tumor fraction of 5.4%. The tumor demonstrated a low tumor mutational burden (TMB) (3.8 mutations/Mb), suggesting a limited likelihood of response to immune checkpoint inhibitors, and no FDA-approved actionable alterations were identified. Although MGMT promoter methylation may indicate potential sensitivity to DNA-damaging agents, its clinical relevance in CRC remains investigational. Pharmacogenomic assessment revealed a normal CYP2D6 activity and an intermediate UGT1A1 metabolizer status, indicating a moderate risk of irinotecan-associated toxicities, particularly neutropenia and diarrhea, thereby warranting close clinical monitoring without precluding its use.

**Figure 3:**
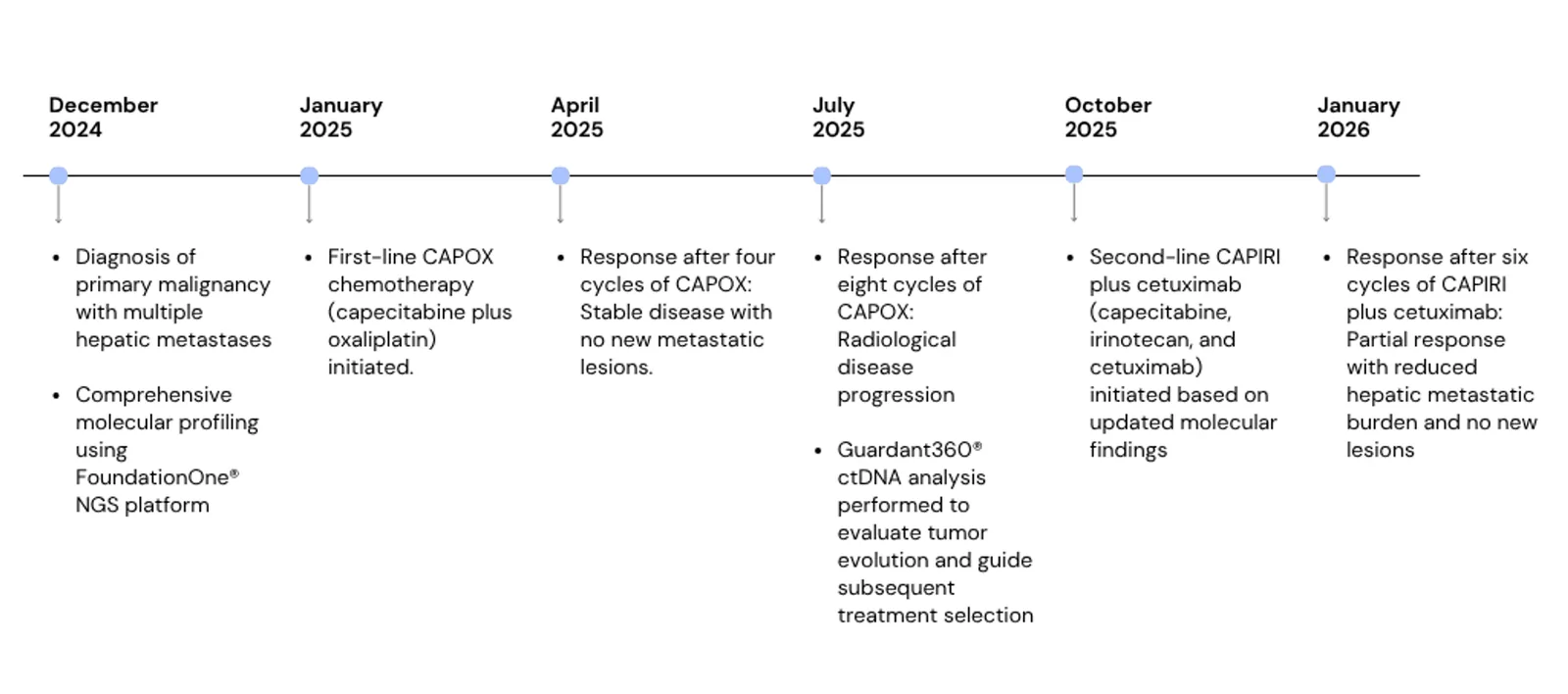
Timeline of the patient's clinical course from diagnosis to the latest follow-up NGS: next-generation sequencing; ctDNA: circulating tumor DNA; CAPOX: capecitabine and oxaliplatin; CAPIRI: capecitabine and irinotecan

Following progression on first-line CAPOX, second-line therapy was initiated in accordance with standard treatment paradigms. In the context of KRAS wild-type disease and the underlying molecular profile suggesting impaired DNA repair capacity, a capecitabine and irinotecan (CAPIRI) regimen was commenced in combination with cetuximab with palliative intent. Irinotecan-based therapy represents the standard second-line approach following oxaliplatin failure, while the presence of MGMT methylation provided a mechanistic rationale for enhanced sensitivity to DNA-damaging agent. The addition of cetuximab was appropriate in the setting of KRAS wild-type disease, further optimizing the therapeutic strategy. Interim response assessment with PET-CT after six cycles demonstrated a partial response (Figure 2D-2F), characterized by a reduction in hepatic metastatic burden with stable disease at the primary site and no evidence of new metastatic lesions. Following the observed partial response, the case was re-evaluated in a multidisciplinary tumor board to assess the feasibility of curative-intent resection. Despite the favorable radiological response, the extent and distribution of metastatic disease remained unsuitable for surgical resection, and systemic therapy with palliative intent was continued. Figure 3 provides a comprehensive timeline of the patient's clinical course, including key diagnostic, molecular, therapeutic, and radiological milestones.

At the most recent follow-up, the patient had completed 10 cycles of CAPIRI-based therapy and continued to maintain an ECOG performance status of 1, with ongoing treatment and supportive care.

## Discussion

This case exemplifies the increasing sophistication required in therapeutic decision-making for mCRC, particularly in patients with a prior history of malignancy where diagnostic ambiguity may confound initial clinical judgment. A fundamental consideration in such scenarios is the accurate distinction between metastatic recurrence of a prior cancer and the emergence of a second primary tumor, as this determination critically directs both staging and treatment strategy. In the present case, comprehensive immunohistochemical characterization demonstrating a CK20⁺/CDX2⁺/SATB2⁺ and CK7⁻/GATA3⁻ phenotype was pivotal in establishing a colorectal origin, thereby enabling appropriate, disease-specific management. The patient's clinical course was marked by aggressive metastatic dissemination with predominant hepatic involvement, a hallmark of advanced CRC, and early progression on oxaliplatin-based chemotherapy (CAPOX), underscoring the well-recognized biological heterogeneity and adaptive resistance mechanisms inherent to mCRC.

Following progression on first-line oxaliplatin-based chemotherapy, second-line treatment was selected in accordance with contemporary biomarker-guided management of metastatic CRC. In the setting of KRAS wild-type disease, the addition of cetuximab to an irinotecan-based regimen represented an evidence-based therapeutic strategy. Although this treatment sequence reflects current standards of care, the patient's favorable response illustrates the continued clinical value of biomarker-directed therapy and provides the foundation for considering additional molecular determinants of treatment response. Beyond conventional clinicopathological determinants, this case highlights the value of comprehensive molecular profiling in refining therapeutic strategies and identifying biologically relevant vulnerabilities. The tumor harbored canonical APC and TP53 alterations, consistent with the chromosomal instability pathway of colorectal carcinogenesis. While these alterations contribute to tumor progression, they do not intrinsically confer resistance to EGFR-targeted therapy and are frequently observed in tumors that remain responsive to anti-EGFR treatment in the absence of RAS/RAF alterations [14]. In addition, microsatellite stability and low PD-L1 expression suggested a limited likelihood of benefit from immune checkpoint inhibitors, supporting the selection of chemotherapy combined with targeted therapy [7].

A notable finding in this case was the identification of MGMT promoter methylation by ctDNA analysis. MGMT is a key DNA repair enzyme, and promoter methylation results in epigenetic silencing with impaired DNA repair capacity, a mechanism classically associated with enhanced sensitivity to alkylating agents such as temozolomide [15]. However, beyond its established role in predicting response to alkylating chemotherapy, MGMT deficiency may serve as a broader surrogate marker of compromised DNA damage response (DDR) and heightened genomic vulnerability.

Although MGMT promoter methylation provides a biological rationale for the use of temozolomide, its role in mCRC remains investigational and is currently supported by limited clinical evidence [16-19]. Consequently, temozolomide has not been incorporated into routine second-line treatment algorithms for mCRC. In contrast, following progression on oxaliplatin-based therapy, irinotecan-based chemotherapy in combination with anti-EGFR therapy for RAS wild-type tumors represents an established, guideline-supported standard of care. Therefore, CAPIRI plus cetuximab was considered the most appropriate therapeutic strategy in the present case, with MGMT promoter methylation providing an additional biological rationale for enhanced irinotecan sensitivity. Within this molecular context, the selection of irinotecan gains additional biological plausibility. Irinotecan exerts its antitumor activity through topoisomerase I inhibition, resulting in DNA damage that ultimately triggers tumor cell death [20]. Its efficacy is therefore influenced by the tumor's capacity to recognize and repair DNA damage. In tumors with impaired DNA repair, such as those exhibiting MGMT promoter methylation, persistence of irinotecan-induced DNA lesions may enhance cytotoxicity [21]. Although MGMT promoter methylation has not been validated as a predictive biomarker for irinotecan response in CRC, emerging evidence suggests that epigenetic defects in DNA repair pathways may increase susceptibility to DNA-damaging agents [21]. This concept is consistent with the broader paradigm of precision oncology, in which defects in DNA repair pathways guide therapeutic selection. Accordingly, MGMT promoter methylation may represent an additional biologically informed marker supporting irinotecan-based therapy, with the favorable response observed in this case providing hypothesis-generating clinical evidence.

Nevertheless, the clinical utility of ctDNA methylation assays remains limited by the need for further analytical validation, inter-platform reproducibility, and standardized clinical thresholds. Furthermore, the favorable treatment response observed in this case cannot be attributed solely to ctDNA-guided therapeutic selection but likely reflects the combined effects of evidence-based second-line CAPIRI therapy, EGFR-targeted treatment in the setting of KRAS wild-type disease, and the potential contribution of MGMT promoter methylation as a marker of impaired DNA repair. Collectively, this case highlights the potential clinical value of integrating real-time ctDNA-based molecular profiling with established therapeutic algorithms to support individualized treatment strategies in mCRC. Although the role of MGMT promoter methylation in predicting irinotecan sensitivity remains investigational, this case supports its further evaluation as a hypothesis-generating biomarker that may complement established molecular stratification in future prospective studies.

## Conclusions

This case underscores the importance of a biomarker-driven approach in mCRC, particularly in distinguishing second primary malignancies from recurrence. The favorable response to CAPIRI plus cetuximab highlights the value of established biomarker-guided treatment sequencing, while the identification of MGMT promoter methylation by ctDNA provides a biologically plausible, hypothesis-generating rationale for the observed response. Further prospective studies are warranted to determine whether MGMT promoter methylation has predictive value for irinotecan-based therapy and to define its role in personalized treatment strategies.
